# PAK4 in metabolic diseases: regulation by nutrient signals and therapeutic implications

**DOI:** 10.1038/s12276-026-01645-y

**Published:** 2026-02-25

**Authors:** In Hyuk Bang, Byung-Hyun Park, Eun Ju Bae

**Affiliations:** 1 Department of Biochemistry and Molecular Biology, Jeonbuk National University, Jeonju, Republic of Korea; 2 Graduate School of Medical Science and Engineering, Korea Advanced Institute of Science and Technology, Daejeon, Republic of Korea; 3 School of Pharmacy and Institute of New Drug Development, Jeonbuk National University, Jeonju, Republic of Korea

**Keywords:** Endocrine system and metabolic diseases, Biochemistry, Translational research

## Abstract

Here we highlight recent advances in understanding the regulatory role of p21-activated kinase 4 (PAK4), the prototypical group II PAK family member, in metabolic diseases. It also briefly notes the contributions of the group I member PAK1 in metabolic tissues. Activation of PAK4 is mediated by upstream Ras-related small GTPases such as Cdc42 and Rac1. In addition to this classical mechanism, post-translational modifications triggered by growth factors and hormonal signals are now recognized as key determinants of PAK4 activity and expression. Notably, phosphorylation-dependent ubiquitination followed by proteasomal degradation—initiated by changes in cellular energy availability—has emerged as an important mechanism regulating PAK4 protein stability. PAK4, in turn, phosphorylates a broad range of intracellular signaling proteins and transcriptional regulators, thereby orchestrating communication among the liver, adipose tissue and skeletal muscle. Accumulating evidence indicates that aberrant overexpression of PAK4 contributes to the progression of metabolic diseases, whereas reduced PAK4 activity may provide protective benefits. These insights collectively support the therapeutic potential of targeting PAK4 in obesity, type 2 diabetes and metabolic dysfunction-associated steatotic liver disease. Moreover, recognition of PAK4’s kinase-independent scaffold functions has stimulated the development of PAK4-targeted protein degraders, expanding therapeutic opportunities.

## PAK4 regulation links metabolism and disease progression

This review article summarizes current evidence supporting PAK4 as an important regulator of metabolic homeostasis and disease. Researchers have found that PAK4 overexpression or sustained activation in adult tissues is closely linked to the progression of metabolic disorders, including obesity, metabolic dysfunction-associated steatotic liver disease and type 2 diabetes. Studies using genetic and pharmacological approaches demonstrate that PAK4 modulates lipid and glucose metabolism through key signaling pathways involving HSL, FABP4, AMPK and NCoR1. Notably, PAK4 inhibition consistently improves metabolic parameters in experimental models, highlighting PAK4 as a promising therapeutic target for metabolic diseases. Future studies should focus on the development and optimization of selective PAK4 inhibitors to enhance therapeutic efficacy in these conditions.

This summary was initially drafted using artificial intelligence, then revised and fact-checked by the author.

## Introduction

**Fig. 1:**
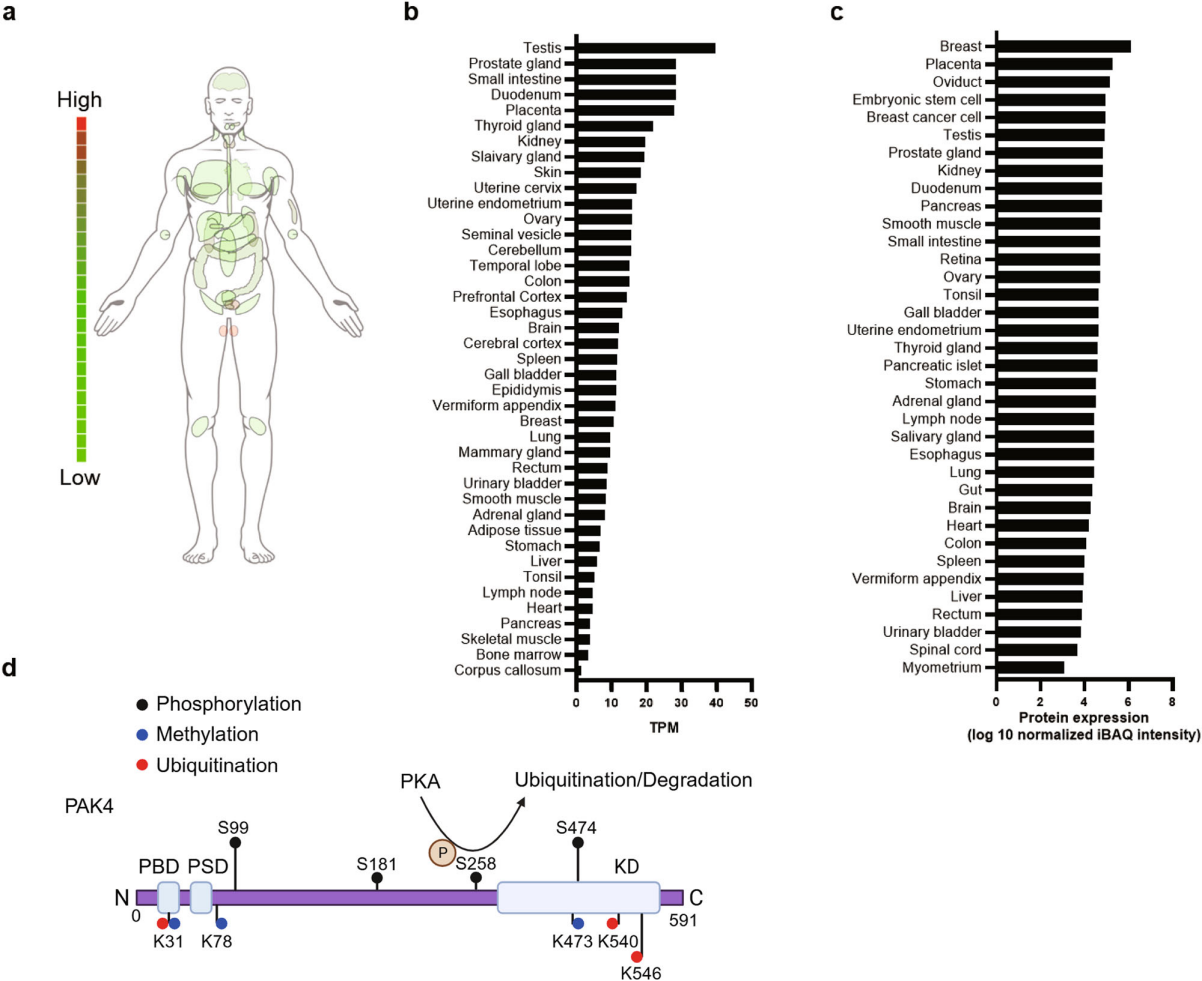
PAK4 expression profiles and schematic overview of its post-translational modification sites. **a** Anatomical distribution of *PAK4* mRNA expression across normal human organs. **b** Normalized expression values of *PAK4* mRNA transcripts retrieved from ProteomicsDB (https://www.proteomicsdb.org). **c** Quantitative proteomics data showing PAK4 protein abundance across major human tissues. **d** The domain structure and major modification sites of PAK4 are illustrated. The Rho GTPases—Cdc42 and Rac1—bind to the PBD. Phosphorylation of Ser474, a marker of PAK4 kinase activity, occurs via autophosphorylation following the conformational change induced by the release of bound Rho GTPase. In addition, PKA-mediated phosphorylation of PAK4 at Ser258 initiates ubiquitination at three lysine residues (K31, K540 and K546), leading to its subsequent proteasomal degradation. TPM transcripts per million, iBAQ intensity-based absolute quantification, PBD p21-binding domain, PSD pseudosubstrate domain, KD kinase domain.

p21-activated kinases (PAKs) are a family of serine/threonine kinases that function downstream of the small GTPases Cdc42 and Rac1^1,2^. Based on their structural features and regulatory mechanisms, PAKs are classified into two groups: group I (PAK1–PAK3) and group II (PAK4–PAK6). Among these, PAK1 and PAK4 have been most extensively studied, initially recognized for their involvement in cytoskeletal regulation and the promotion of cancer cell proliferation, migration and metastasis^3^. The *Pak4* gene is widely expressed across multiple tissues, with particularly high levels in the prostate, testis and kidney^4^. By contrast, PAK4 expression in major metabolic organs—such as the liver, adipose tissue and skeletal muscle—is relatively low under normal condition (Fig. 1a–c) but increases markedly in these tissues under metabolic disease states^5–7^. PAK4 is indispensable for embryonic development in mice, as global knockout (KO) models exhibit embryonic lethality by day E11.5, probably due to cardiac defects^8^. More recent studies employing tissue-specific *Pak4*-KO mice—targeting adipocytes, preadipocytes, hepatocytes and skeletal muscle cells—together with pharmacological PAK4 inhibitors, have revealed a strong association between PAK4 activity and metabolic diseases such as obesity, type 2 diabetes (T2D) and metabolic dysfunction-associated steatotic liver disease (MASLD), as well as with oxidative stress and tissue regeneration^5–7,9–12^. These findings suggest that PAK4 inhibition may represent a promising therapeutic strategy. In the following sections, this review article synthesizes current knowledge on the roles of PAK4 in metabolic regulation, with a focus on adipose tissue, liver and skeletal muscle, while also considering the broader contributions of PAK1 signaling to metabolic processes.

## Structure and activation model of PAK4

### Structural characteristics of PAK4

Members of the PAK family share a conserved modular architecture comprising an N-terminal regulatory region and a C-terminal serine/threonine kinase domain. The regulatory segment typically contains a p21-binding domain (PBD) that mediates interactions with the Rho GTPases Cdc42 and Rac, as well as an autoinhibitory domain (AID) that constrains kinase activity (Fig. 1d). On the basis of domain composition and regulatory properties, PAKs are classified into two subgroups. Group I members (PAK1-3) possess both a canonical PBD and AID, with the AID forming inhibitory dimers that suppress the catalytic domain. Group II members (PAK4-6), by contrast, harbor a PBD followed by an AID-like sequence or a pseudosubstrate domain (PSD), which acts as a distinct inhibitory module.

PAK4 is the most extensively investigated member of the group II PAK family. The PBD of PAK4 demonstrates high-affinity binding to Cdc42 but only weak interactions with Rac, underscoring its preferential regulation by Cdc42. The PSD plays a central role in maintaining the kinase in an inactive state by mimicking substrate residues and blocking access to the catalytic cleft, thereby imposing autoinhibition until appropriate signals are received.

### Activation model of PAK4 from structural and signaling perspectives

Multiple structural models have been proposed to explain how PAK4 transitions from an inactive to an active conformation. Baskaran and colleagues demonstrated that although PAK4 is constitutively phosphorylated at Ser474, kinase activity remains repressed by PSD-mediated autoinhibition, which is alleviated upon Cdc42 binding^13^. By contrast, Ha et al. proposed that the PSD occupies the substrate-binding pocket and that association with Cdc42 induces conformational rearrangements that permit SH3 domain-containing cofactors to further stimulate kinase activity^14^. Structural analyses revealed an extensive hydrogen-bonding network between the PSD and catalytic cleft, accounting for the strength of autoinhibition, while also identifying direct interactions between Cdc42 and the kinase domain that form a ternary complex and partially destabilize the inhibitory conformation.

In addition to Cdc42-dependent mechanisms, a number of signaling pathways have been implicated in PAK4 activation. Hepatocyte growth factor can stimulate PAK4 through phosphoinositide 3-kinase (PI3K) signaling to regulate cytoskeletal remodeling^15^. Protein kinase C (PKC) phosphorylates PAK4 at Ser99 and Ser181, facilitating its recruitment to the leading edge of migrating cells and promoting downstream activation of LIM domain kinase 1 (LIMK1) and β-catenin^16^. Protein kinase D1 (PKD1) has also been shown to phosphorylate Ser99, thereby enabling 14–3–3 protein binding and the formation of a PAK4–LIMK–PKD1 complex that orchestrates cofilin activity and actin dynamics^17^. Collectively, these findings highlight the multifaceted nature of PAK4 activation, integrating both small GTPase and kinase-mediated inputs.

### Transcriptional regulation of the *Pak4* gene

Transcriptional control adds an additional layer of regulation to PAK4 signaling. In mouse embryonic stem cells, Nanog directly binds to the *Pak4* promoter, initiating a Nanog–PAK4–Akt signaling axis that is critical for the maintenance of pluripotency^18^. PAK4 expression is also responsive to hypoxic conditions. During hepatic ischemia–reperfusion injury, stabilization of hypoxia-inducible factor 1α (HIF1α) promotes its recruitment to hypoxia response elements (HREs) within the *Pak4* promoter, leading to transcriptional upregulation. PAK4, in turn, phosphorylates the transcription factor nuclear factor erythroid 2-related factor 2 (Nrf2) at Thr369, thereby dampening antioxidant defense pathways^10^. In skeletal muscle, *Pak4* expression can also be modulated by glucocorticoids, as dexamethasone was shown to activate transcription via a glucocorticoid response element (GRE) in the promoter; deletion of this GRE abrogates the effect, implicating PAK4 in glucocorticoid-driven muscle atrophy^19^. Together, these structural, biochemical and transcriptional studies provide a mechanistic framework for understanding how PAK4 integrates upstream signals to regulate diverse cellular processes, setting the stage for its roles in metabolic and pathological contexts.

## Post-translational modification of PAK4

### Phosphorylation

PAK4 activation is triggered by Cdc42 engagement with its p21-binding domain, which releases an N-terminal pseudosubstrate constraint and permits autophosphorylation that enhances catalytic activity^14^. This modification occurs at Ser474 within the C-terminal activation loop, a site critical for both full catalytic activation and structural stabilization of the protein^20^. In addition to autophosphorylation, protein kinase A (PKA) can phosphorylate PAK4 at Ser474, leading to activation of the cAMP response element-binding protein (CREB) and promoting prostate cancer progression^21^. These findings suggest the existence of a positive feedback loop between PKA and PAK4 in cancer contexts. Conversely, our recent work demonstrated that PKA activation—via isoproterenol stimulation—induces phosphorylation of PAK4 at Ser258 in adipocytes, which facilitates its ubiquitination and subsequent proteasomal degradation^7^ (Fig. 1d). Pharmacological inhibition of PKA with H89 or Rp-cAMP, as well as a phosphorylation-deficient Ser258-to-alanine mutation (S258A), completely prevented PAK4 degradation, indicating that PKA also functions as a negative regulator of PAK4 stability. This inhibitory role was further confirmed in primary murine hepatocytes, where glucagon or forskolin-mediated activation of PKA destabilized PAK4 protein^5^. Collectively, these findings reveal a context-dependent mode of regulation in which PKA enhances PAK4 kinase activity in prostate cancer cells, whereas in metabolic tissues such as adipocytes and hepatocytes, PKA signaling instead downregulates PAK4 abundance. Although the underlying mechanisms remain to be clarified, cell type-specific differences may account for these contrasting outcomes. Importantly, direct interaction between the PKA catalytic subunit and PAK4 has been observed across diverse cell types^7,21^. Beyond PKA, PAK4 is also phosphorylated by PKD1 at Ser99, a modification that alters its subcellular localization and may indirectly influence protein stability and downstream signaling^17^.

### Ubiquitination

Regulation of PAK4 stability via ubiquitination represents a critical post-translational control mechanism in response to nutrient status. Our previous studies in adipocytes and hepatocytes demonstrated that PAK4 protein levels are rapidly diminished during fasting and restored upon refeeding, indicating dynamic, context-dependent regulation^5,7^. Consistently, Kim et al. reported that PAK4 is subject to ubiquitination, which targets it for proteasomal degradation and reduces its half-life in colon cancer cells^22^. In this context, SH3RF2, a member of the SH3 domain-containing RING finger (SH3RF) protein family, functions as an E3 ligase scaffold that binds PAK4 and prevents its ubiquitination, thereby stabilizing the protein. Similarly, Rac1 activity was shown to protect PAK4 from ubiquitination, and inhibition of Rac1 promoted PAK4 degradation, whereas Rac1 overexpression enhanced protein stability in ovarian cancer cells^23^.

Building on these findings, our recent work demonstrated that nutrient-sensing pathways involving cAMP–PKA and Sirt1 induce PAK4 ubiquitination during fasting^5,7^. PKA activation decreased PAK4 protein abundance without altering mRNA levels, highlighting post-translational control. Through co-immunoprecipitation, in vitro kinase assays and liquid chromatography–tandem mass spectrometry (LC–MS/MS) analysis, we established that PKA directly phosphorylates PAK4 at Ser258, thereby facilitating its ubiquitination and subsequent proteasomal degradation in both adipocytes and hepatocytes. Inhibition of PKA—via refeeding, insulin stimulation or pharmacological blockade—effectively restored PAK4 protein levels. Site-directed mutagenesis further identified lysine residues Lys31, Lys540 and Lys546 in PAK4 as essential ubiquitination sites mediating its degradation^5^ (Fig. 1d). Moreover, comprehensive LC–MS/MS analysis of PAK4 immunoprecipitates combined with functional validation pinpointed ubiquitin-conjugating enzyme E2 O (UBE2O) and mouse double minute 2 homolog (MDM2) as key E3 ligases mediating PKA-induced ubiquitination, as silencing either enzyme markedly attenuated PAK4 degradation^7^.

### Acetylation

Acetylation also contributes to PAK4 regulation. Immunoprecipitation–western blot analysis with a pan-acetyl-lysine antibody revealed that PAK4 is highly acetylated in hepatocytes under fed conditions^5^. Exposure to ketogenesis precursors—such as palmitate, octanoate or β-hydroxybutyrate (βOHB)—increased Sirt1 expression and consequently promoted PAK4 destabilization, whereas Sirt1 knockdown or pharmacological inhibition led to PAK4 stabilization. These findings indicate that Sirt1-mediated deacetylation facilitates PAK4 ubiquitination, thereby contributing to its suppression during fasting^5^.

### Methylation

In addition to phosphorylation, ubiquitination and acetylation, PAK4 can undergo mono-methylation mediated by SET domain-containing 6 (SETD6), a lysine methyltransferase^24^. SETD6 targets lysine residues Lys31, Lys78 and Lys473 (Fig. 1d), promoting PAK4’s interaction with β-catenin and enhancing Wnt/β-catenin signaling activity. Although this modification does not directly trigger PAK4 degradation, it modulates protein function and chromatin interactions, thereby indirectly influencing PAK4 stability under conditions such as cellular stress.

## PAK1 in metabolic diseases

Although PAK1 is the most extensively studied member of the PAK family, its functions in metabolic tissues have remained largely unexplored, with only a few reports beginning to delineate its role. PAK1 is highly expressed in the brain, skeletal muscle and spleen^4^, and its diverse physiological and pathological roles have been comprehensively reviewed^25^. Importantly, PAK1 is essential for maintaining normal myocardial function, and its inhibition can lead to severe cardiac toxicity^26^. In the context of energy metabolism, PAK1 has been implicated in the regulation of skeletal muscle, liver and pancreatic β-cell function.

### Role of PAK1 in skeletal muscle insulin sensitivity

A small GTPase Rac1 is critical for insulin-stimulated glucose transporter type 4 (GLUT4) translocation to the plasma membrane^27^. PAK1, as a downstream effector of Rac1, is likewise essential for GLUT4 trafficking and insulin-mediated glucose uptake in skeletal muscle^26,28^. This dependency is consistent with the fact that insulin activates PAK1 through PI3K-mediated phosphorylation^29^, and PAK1 is indispensable for actin cytoskeleton remodeling required for glucose uptake^30^. Skeletal muscle-specific *Pak1*-KO mice exhibit insulin resistance and impaired glucose tolerance^31^. In humans with T2D, PAK1 protein—but not mRNA—is reduced in skeletal muscle^31^, contrasting with PAK4, which is upregulated and contributes to impaired glucose uptake in T2D muscle^6^.

### PAK1 in liver function and disease

The role of PAK1 in liver physiology and disease extends beyond its implication in hepatocellular carcinoma (HCC). Given that PAK1 is activated by insulin, its involvement in hepatocyte function and systemic glucose homeostasis is expected. Zeng et al. demonstrated that insulin stimulates PAK1 through Thr423 phosphorylation in hepatocytes and that insulin-induced carbohydrate-response element-binding protein (ChREBP) expression was dependent on PAK1^32^. Specifically, hepatocytes lacking PAK1 exhibited impaired ChREBP induction, ERK activation and lipogenic gene expression in response to insulin, whereas Akt phosphorylation remained unaffected. These findings suggest that the insulin–PAK1–ERK signaling axis may underlie selective hepatic insulin resistance, wherein insulin-driven lipogenesis remains preserved while insulin-mediated suppression of gluconeogenesis becomes resistant under obesity-associated insulin resistance.

During the progression of liver fibrosis, PAK1 in hepatic stellate cells (HSCs) plays a pivotal role. While PAK1 expression is minimal in quiescent HSCs, it is markedly induced upon activation^33^. Consistent with this, we also observed increased PAK1 expression in activated HSCs but not in whole liver tissue from carbon tetrachloride (CCl₄)-induced cirrhotic mice. By contrast, PAK4 was significantly upregulated in both activated HSCs and liver tissues from cirrhotic mice (unpublished results). Moreover, pharmacological inhibition of PAK1 with IPA-3 (2,2′-dihydroxy-1,1′-dinaphthyldisulfide) attenuated HSC activation and reduced fibrosis in both CCl₄- and bile duct ligation-induced models, despite no changes in conventional liver injury markers such as ALT and bilirubin^33^. These findings suggest that while PAK1 inhibition shows promise in modulating fibrogenic responses, its therapeutic potential in liver disease requires further validation.

### PAK1 in pancreatic β-cells

PAK1 also plays a critical role in pancreatic β-cells, where it supports diet-induced β-cell mass expansion and survival in both murine and human islets^34^. Mechanistically, PAK1 regulates F-actin dynamics to facilitate insulin granule exocytosis. Notably, PAK1 protein levels are decreased in β-cells from individuals with T2D, implicating its deficiency in impaired insulin secretion and β-cell dysfunction^35^.

Beyond pancreatic β-cells, gut endocrine L cells also contribute to PAK1’s role for insulin secretion and glucose disposal. In proglucagon (*Gcg*)-expressing intestinal cells, insulin activates PAK1 via Thr423 phosphorylation, which enhances proglucagon expression and increases the production of the incretin hormone glucagon-like peptide-1^36^. Conversely, *Pak1* deletion or pharmacological inhibition with the allosteric PAK1 inhibitor IPA-3 abolishes these effects, resulting in the impaired glucose tolerance.

## PAK4 in disease pathogenesis

### Overexpression and clinical correlations

PAK4 is frequently overexpressed across a variety of solid tumors, where its upregulation drives aggressive phenotypes^37,38^. In breast and ovarian cancers, elevated PAK4 promotes cell motility, invasiveness and therapy resistance, whereas in oral squamous cell carcinoma and cervical cancer, high PAK4 expression is associated with larger tumor size, deeper tissue invasion, advanced clinical stage and poorer overall survival. These observations underscore PAK4 as a recurrent oncogenic driver and highlight its potential as both a prognostic marker and a therapeutic target.

Beyond oncology, emerging evidence indicates that PAK4 overexpression also contributes to metabolic disease pathogenesis. Recent studies demonstrated that PAK4 is upregulated in tissues affected by obesity, T2D and MASLD, with protein levels correlating with disease severity in both murine models and human patients. For instance, in visceral adipose tissue, PAK4 protein levels were increased in obese mice and obese individuals, showing a positive correlation with body mass index and waist circumference^7^. Notably, *Pak4* mRNA levels remained unchanged, suggesting that the upregulation results from impaired protein degradation. Under normal conditions, PKA-mediated phosphorylation at Ser258 promotes PAK4 ubiquitination and proteasomal degradation, but reduced PKA activity in obesity leads to PAK4 accumulation. Similarly, in the liver and skeletal muscle, diminished PKA or Sirt1 signaling prevents PAK4 degradation, contributing to elevated protein levels observed in MASLD and T2D^5,6^.

Collectively, these findings indicate that dysregulated PAK4 not only drives tumor progression but also exacerbates metabolic disease, emphasizing its potential as a therapeutic target in diverse pathological contexts.

### Adipocyte physiology in lipolysis, browning and adipogenesis

Adipocytes are specialized for energy storage in lipid droplets (LDs), which primarily consist of triglycerides and cholesteryl esters. Once considered inert reservoirs, LDs are now recognized as dynamic organelles that regulate lipid storage, mobilization and metabolic signaling. LDs are coated with perilipins, which in the fed state sequester comparative gene identification 58 (CGI58) to prevent activation of adipose triglyceride lipase. During fasting, catecholamine-β-adrenergic signaling activates PKA, leading to perilipin phosphorylation, release of CGI58, adipose triglyceride lipase activation and concurrent phosphorylation of hormone-sensitive lipase (HSL), which together with monoacylglycerol lipase orchestrates the lipolytic cascade. Lipolysis also depends on the coordinated interaction between lipases and cofactors, such as fatty acid binding protein 4 (FABP4, also known as aP2 or A-FABP). FABP4, expressed in adipocytes and macrophages, promotes lipolysis and serves as a cardiometabolic biomarker by binding free fatty acids and facilitating their intra- and extracellular transport^39^. Genetic deletion or inhibition of FABP4 impairs HSL-mediated lipolysis, underscoring its essential role. Despite being PKA-dependent, the regulation of intracellular FABP4 trafficking has remained incompletely understood.

Recent studies have identified PAK4 as a key regulator of adipocyte lipolysis through direct phosphorylation of FABP4^7^. Phosphoproteomic analyses of epididymal adipose tissue from fasted adipocyte-specific *Pak4*-KO mice, combined with co-immunoprecipitation, revealed FABP4 as a PAK4-interacting partner. LC–MS/MS identified three threonine residues—Thr8, Thr124 and Thr126—phosphorylated by PAK4, with functional assays pinpointing Thr126 as the critical site disrupting the FABP4–HSL interaction. Overexpression of a phospho-mimetic mutant (Thr126-to-aspartate mutation, T126D) markedly reduced oxidative phosphorylation, as indicated by lower oxygen consumption and reduced oxidative phosphorylation protein expression. Similar outcomes were observed in C57BL/6 adipocytes ectopically overexpressing PAK4, supporting the concept that fatty acids liberated during lipolysis are not only exported to peripheral tissues but can also be oxidized autonomously within adipocytes.

**Fig. 2:**
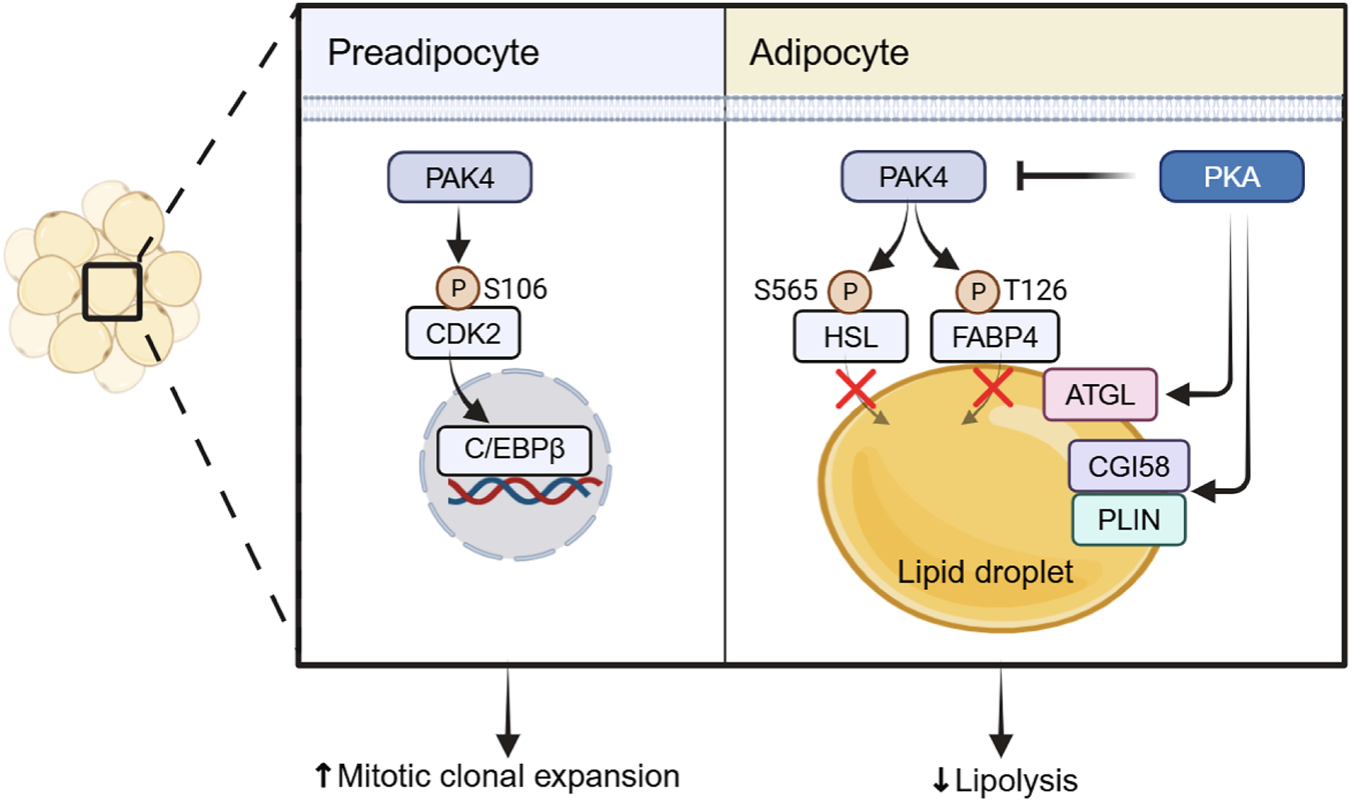
Mechanisms by which PAK4 regulates adipogenesis and lipolysis in preadipocytes and adipocytes. In preadipocytes, PAK4 is essential for mitotic clonal expansion by phosphorylating CDK2, thereby activating C/EBPβ. In adipocytes, upregulation of PAK4 in obesity enhances phosphorylation of HSL at Ser565 and FABP4 at Thr126, which suppresses HSL activity and disrupts FABP4–HSL interaction, respectively. Consequently, PAK4 inhibits lipolysis and adipocyte browning, thereby promoting obesity. ATGL adipose triglyceride lipase; CGI58 comparative gene identification-58; PLIN perilipin.

PAK4 also inhibits lipolysis via direct phosphorylation of HSL at Ser565, a site previously reported to be targeted by AMP-activated protein kinase (AMPK)^40,41^. Phosphorylation at Ser563 or Ser660 promotes HSL activation, whereas Ser565 phosphorylation inhibits it. Together with FABP4 modulation, this mechanism positions PAK4 as a critical antilipolytic regulator (Fig. 2). Consistent with this, Seigner et al. reported that group II PAKs suppress adipocyte lipolysis by phosphorylating and activating phosphodiesterase 4D, reducing cytosolic cAMP and impairing HSL activity^42^. While this pathway differs mechanistically from PAK4’s direct regulation, it supports the broader role of group II PAKs in limiting lipolysis. Notably, intracellular cAMP levels were unchanged in PAK4-deficient adipocytes, indicating that PKA functions upstream of PAK4, which directly modulates HSL and FABP4. Collectively, these findings establish a PAK4–FABP4 axis as a bona fide determinant of lipolytic regulation.

Beyond stimulating lipolysis, adipocyte-specific PAK4 deficiency promotes adipocyte browning—evidenced by increased beiging in inguinal adipose tissue and activation of brown adipose tissue^7^. These coordinated effects of PAK4 inhibition, namely elevated lipolysis and augmented browning, collectively increase energy expenditure and thereby protect against high-fat diet (HFD)-induced obesity. Whether liberated free fatty acids serve directly as fuel for browning in inguinal or brown adipose tissues remains to be determined.

PAK4 also plays a pivotal role in adipogenesis. In preadipocytes, PAK4 phosphorylates cyclin-dependent kinase 2 (CDK2) at Ser106, a modification required for the transactivation of CCAAT enhancer binding protein beta (C/EBPβ), a transcription factor essential during mitotic clonal expansion^12^. Preadipocyte-specific *Pak4*-KO mice exhibited reduced body weight and fat mass under normal chow conditions, highlighting the importance of PAK4 in adipose tissue development (Fig. 2).

Taken together, these studies indicate that PAK4 exerts dual functions in adipose tissue: promoting adipogenesis in preadipocytes while suppressing lipolysis in mature adipocytes, suggesting that therapeutic targeting of PAK4 could be a potential strategy for obesity prevention and treatment.

### Liver physiology in fatty acid oxidation and ketogenesis

MASLD encompasses a broad spectrum of liver disorders, ranging from simple steatosis to steatohepatitis, fibrosis and cirrhosis, and may ultimately progress to HCC. While prior studies primarily highlighted the oncogenic functions of liver PAK4—promoting HCC development and metastasis via mechanisms including p53 phosphorylation^38^, cell cycle regulation^43^ and enhanced cellular migration and invasion^44^—its role in metabolic regulation beyond cancer remained largely unexplored. Recent our study demonstrates that PAK4 critically modulates fatty acid oxidation and ketogenesis under both physiological conditions, such as fasting and nutritional interventions, including ketogenic diet feeding^5^.

Under normal feeding conditions, hepatic PAK4 protein levels are moderate; however, fasting or ketogenic diet induces rapid suppression, inversely correlating with glucagon and βOHB levels. Conversely, metabolic disease states such as HFD feeding or genetic obesity models (*ob/ob* and *db/db*), display elevated hepatic PAK4 expression. To dissect its metabolic functions, hepatocyte-specific *Pak4*-KO mice were generated. Compared with wild-type controls, *Pak4*-KO mice exhibited enhanced ketogenesis, reduced hepatic and circulating triglycerides and decreased markers of hepatic stress. These phenotypic changes were accompanied by upregulation of key genes involved in fatty acid β-oxidation and ketogenesis, including *Cpt1a*, *Hmgcs2* and *Ppara*, indicating that hepatocyte PAK4 functions as a suppressor of both lipid catabolism and ketone body production.

**Fig. 3:**
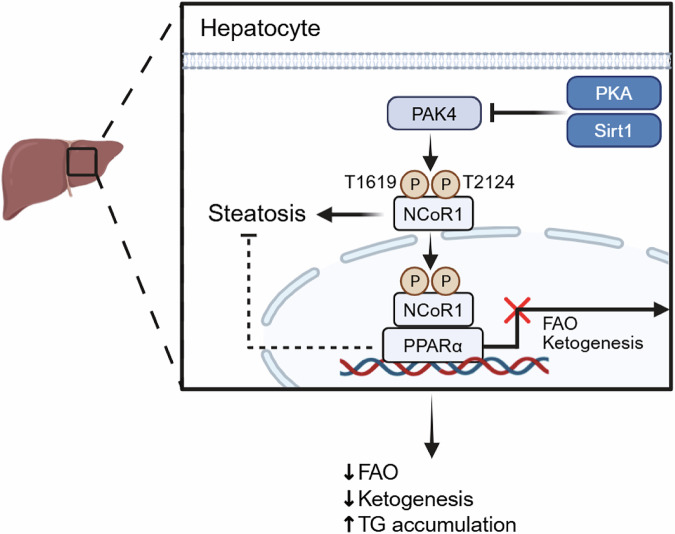
Mechanisms by which PAK4 regulates ketogenesis and fat accumulation in hepatocytes. PAK4-dependent phosphorylation of NCoR1 at Thr1619 and Thr2124 enhances its nuclear retention and interaction with PPARα, thereby repressing PPARα transcriptional activity. As PPARα is a key regulator of fatty acid β-oxidation (FAO) and ketogenesis, this suppression reduces FAO and ketone body production, while promoting triglyceride (TG) accumulation and hepatic steatosis.

Mechanistically, RNA-seq analyses, PhosphoNET predictions and in vitro studies revealed that PAK4 phosphorylates nuclear receptor corepressor 1 (NCoR1) at Thr1619 and Thr2124. This modification enhances NCoR1 nuclear localization and its interaction with peroxisome proliferator-activated receptor alpha (PPARα), thereby repressing PPARα transcriptional activity (Fig. 3). Importantly, this regulation appears selective, as interactions between NCoR1 and liver X receptor alpha (LXRα), or PPARα and p300/SMRT, remained unchanged, although PAK4 overexpression did reduce NCoR1 binding to thyroid hormone receptor beta (THRβ), a key regulator of hepatic lipid metabolism^45,46^.

Functionally, ketone bodies generated during enhanced fatty acid oxidation can suppress tumor growth through mechanisms such as histone deacetylase inhibition^47,48^. Consistent with this, ketogenic diet-fed wild-type mice displayed reduced tumor mass compared with those on a normal chow diet, whereas ketogenic diet-fed *Pak4*-KO mice exhibited further suppression of tumor growth, which correlated inversely with blood and hepatic βOHB levels.

Clinical relevance was further underscored by analyses of tissues of patients with HCC. Elevated PAK4 expression was associated with poorer relapse-free and overall survival, whereas high 3-hydroxy-3-methylglutaryl-CoA synthase 2 (HMGCS2) levels predicted favorable outcomes. Multivariate analyses identified both PAK4 and HMGCS2 as independent prognostic indicators. Tumor tissues displayed increased PAK4, phosphorylated/nuclear NCoR1 and decreased HMGCS2 compared with non-tumorous counterparts, with βOHB levels inversely correlated with PAK4 expression.

Collectively, these findings establish PAK4 as a critical negative regulator of hepatic fatty acid oxidation and ketogenesis, linking its dysregulation to steatosis, tumor progression and poor clinical prognosis. These results position PAK4 as a promising therapeutic target for both MASLD and liver cancer.

### Skeletal muscle insulin resistance and glucose uptake

Building upon observations that PAK4 deficiency in adipocytes and hepatocytes enhances systemic insulin sensitivity, recent studies have explored its potential role in skeletal muscle glucose homeostasis. In obese mice with T2D, skeletal muscle PAK4 protein levels were markedly elevated compared with healthy controls^6^. Muscle-specific deletion of *Pak4* improved systemic insulin sensitivity and glucose tolerance relative to wild-type mice, contrasting with the phenotype observed in *Pak1*-KO mice, which exhibited exacerbated insulin resistance^28,31^. These metabolic improvements in *Pak4*-KO mice were accompanied by activation of the AMPK pathway, as evidenced by increased phosphorylation at the canonical activation site Thr172 and decreased inhibitory phosphorylation at AMPKα2-Ser491 (Ser487 in humans). Consistent with these findings, transcriptomic analyses from the GTEx database revealed a strong association between PAK4 expression and AMPK-related signaling pathways. Furthermore, *Pak4*-KO mice displayed significant increases in both total and plasma membrane GLUT4 expression, independent of nutritional intake, highlighting a direct regulatory role of PAK4 in skeletal muscle glucose handling.

**Fig. 4:**
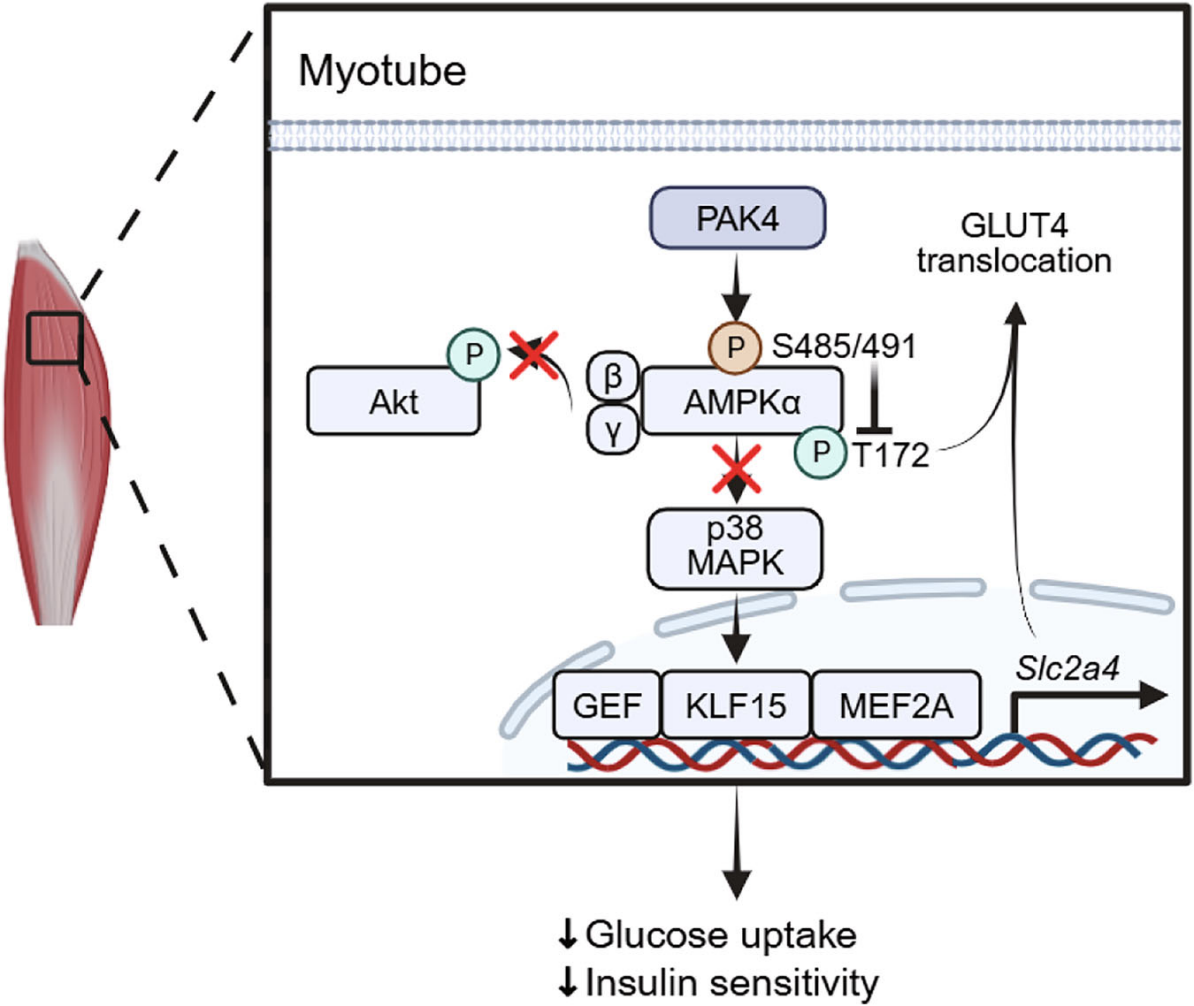
Mechanisms by which PAK4 regulates glucose uptake in skeletal muscle. AMPKα activity is essential for both GLUT4 transcription and its trafficking to the plasma membrane. PAK4 phosphorylates AMPKα1/2 at Ser485/Ser491 while concurrently inhibiting phosphorylation at Thr172, thereby suppressing AMPK activation. GLUT4, glucose transporter type 4.

Mechanistic investigations demonstrated that PAK4 directly interacts with AMPKα2, phosphorylating it at Ser491 while simultaneously inhibiting Thr172 phosphorylation, thereby suppressing overall AMPK activity (Fig. 4). Functional validation studies revealed that expression of a phospho-mimetic AMPKα2-S491D mutant exacerbated insulin resistance, whereas a phospho-deficient AMPKα2-S491A mutant enhanced glucose tolerance. These results identify Ser491 phosphorylation of AMPKα2 as a critical molecular event underlying skeletal muscle insulin resistance.

Importantly, this work provides the first in vivo evidence that AMPKα2-Ser491 phosphorylation drives systemic glucose intolerance. These findings are in line with previous reports indicating that PKD1-mediated phosphorylation of AMPKα2-Ser491 disrupts insulin signaling^49^ and that SGK1-mediated phosphorylation of AMPKα1-Ser485 induces hepatic insulin resistance^50^. While several kinases, including Akt^51–53^, p70 S6 kinase 1^54^, SGK1^50^, glycogen synthase kinase 3^55^, PKD1^49^ or IκB kinase beta^56^ have been implicated in Ser485/491 phosphorylation, PAK4 emerges as an independent kinase targeting this residue. Notably, the deletion of *Pak4* in L6 myotubes attenuated insulin-stimulated AMPKα-Ser485/491 phosphorylation, whereas insulin-stimulated Akt phosphorylation was paradoxically enhanced, suggesting that PAK4 contributes to Akt-mediated AMPKα-Ser485/491 phosphorylation in a context-dependent manner.

Finally, analysis of skeletal muscle biopsies from patients with T2D revealed elevated PAK4 expression and increased AMPKα-S487/491 phosphorylation, accompanied by reduced GLUT4 expression and impaired AMPK/p38 MAPK signaling.

Collectively, these findings establish PAK4 as a bona fide inhibitory kinase of AMPK in skeletal muscle, directly impairing glucose uptake and contributing to insulin resistance, highlighting its potential as a therapeutic target for improving muscle insulin sensitivity.

### Role of PAK4 in ischemia–reperfusion injury

Ischemia–reperfusion remains a principal mechanism of tissue injury under perioperative and critical care conditions, significantly contributing to morbidity and mortality. Organs with high metabolic demand and oxygen consumption, including the liver, heart and kidneys, are particularly susceptible to ischemia–reperfusion, which triggers mitochondrial dysfunction, oxidative stress and subsequent cell death. Emerging evidence indicates that PAK4 functions as a maladaptive regulator in the context of ischemia–reperfusion injury. In hepatocytes, PAK4 directly phosphorylates Nrf2 at Thr369, promoting its nuclear export and subsequent proteasomal degradation^10^. This post-translational modification attenuates Nrf2-dependent antioxidant responses, thereby diminishing the hepatocyte’s capacity to counteract reactive oxygen species-mediated injury. Conversely, both genetic ablation and pharmacological inhibition of PAK4 preserve Nrf2 activity, enhancing hepatoprotection and mitigating ischemia–reperfusion-induced tissue damage.

## PAK4 inhibitors and future therapeutic directions

Recent studies underscore the therapeutic potential of targeting PAK4—primarily as an anticancer strategy—using both small-molecule inhibitors and proteolysis-targeting chimera (PROTAC)-based degraders. Several PAK4 inhibitors have been developed, including PF-3758309 (a pan-PAK inhibitor)^57^, GNE-2861 (a group II PAK inhibitor)^58^, the dual PAK4/NAMPT inhibitor KPT-9274^59^ and CZh-226, a selective PAK4 inhibitor with limited oral bioavailability^60^. PF-3758309 was discontinued in phase I clinical trials due to unfavorable pharmacokinetic properties, whereas KPT-9274—an oral dual PAK4/NAMPT inhibitor—is currently being evaluated in a phase I trial for advanced solid tumors and non-Hodgkin’s lymphoma. So far, no highly selective PAK4 inhibitor has progressed to clinical application, underscoring the need for next-generation, truly PAK4-selective therapeutics with improved oral bioavailability. ND201651 was developed as a selective, orally bioavailable PAK4 inhibitor^10^ and has been evaluated for its efficacy in various mouse models of metabolic disease^5–7,19^.

**Fig. 5:**
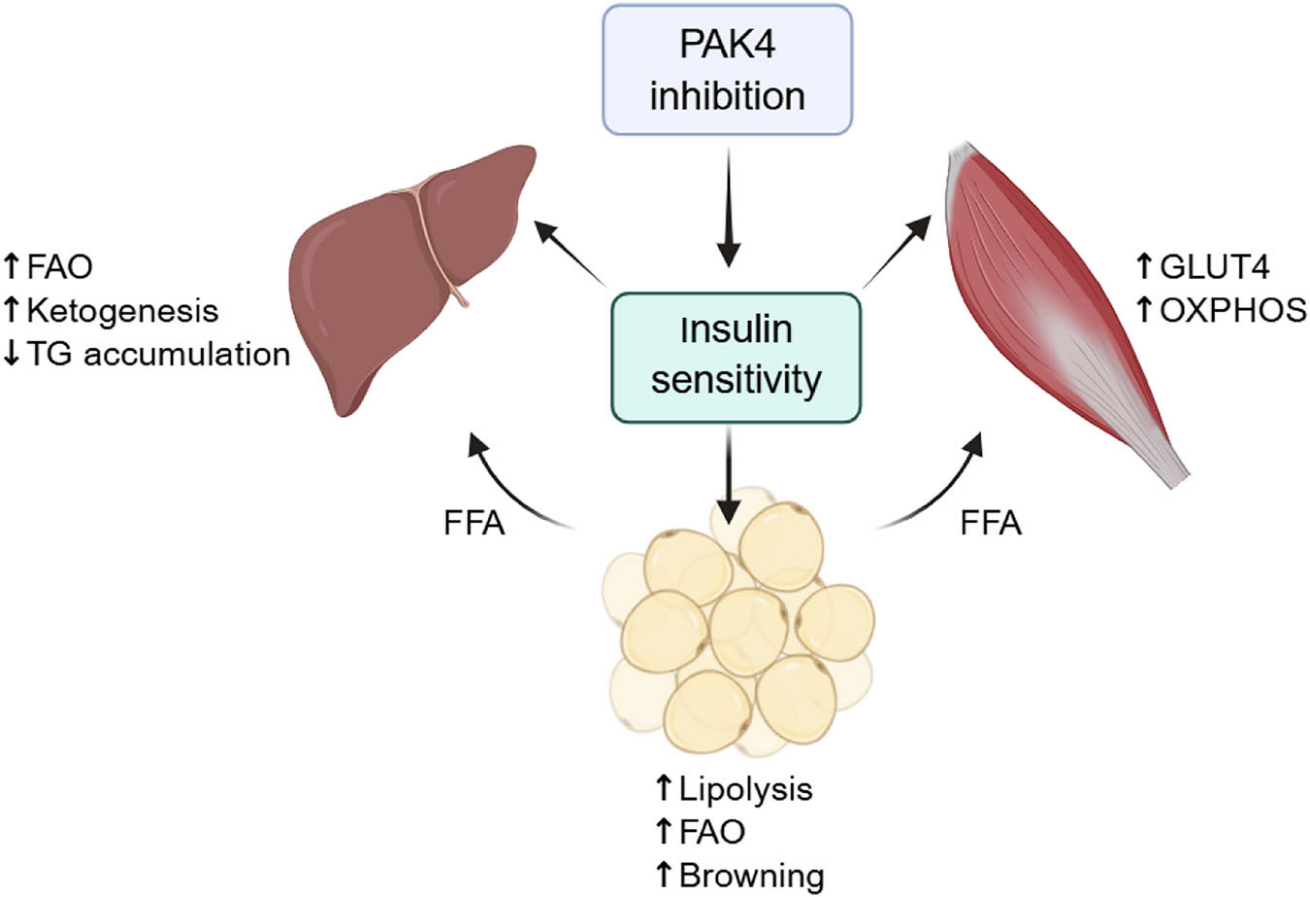
Proposed mechanism by which PAK4 inhibition could serve as a therapeutic strategy against metabolic diseases. Pharmacological inhibition of PAK4, either through small-molecule inhibitors or PROTAC-based protein degraders, holds promise for anti-obesity, antidiabetic and antisteatotic efficacy. FAO fatty acid β-oxidation, TG triglyceride, FFA free fatty acid, OXPHOS oxidative phosphorylation.

In obese mouse models, administration of ND201651 led to significant reductions in body weight without altering food intake or physical activity, attenuated adipocyte hypertrophy and macrophage infiltration and improved glucose and insulin tolerance^7^. ND201651 treatment upregulated genes involved in fatty acid oxidation, including *Cpt1a*, *Acox1* and *Hmgcs2*, and reduced hepatic lipid accumulation under both HFD and ketogenic diet feeding conditions^5^. In skeletal muscle, ND201651 improved glucose tolerance, enhanced GLUT4 expression and membrane localization and activated AMPK signaling. These effects were recapitulated in vitro and were absent in *Pak4*-KO mice, confirming target specificity^6^. Collectively, these findings indicate that pharmacological inhibition of PAK4 confers broad metabolic benefits across adipose tissue, liver and skeletal muscle (Fig. 5).

More recently, PAK4-selective protein degraders have expanded the therapeutic landscape. The first to be reported was a peptide-based PAK4 PROTAC, which showed significant tumor inhibitory activity in renal carcinoma when administered intraperitoneally every other day for 3 weeks^61^. A second PAK4-targeting PROTAC demonstrated efficacy in suppressing lung tumor metastasis following intravenous administration for two weeks, further supporting PAK4 degradation as a promising therapeutic strategy^62^. Building upon ND201651, we recently synthesized a novel PAK4-selective PROTAC, SJ-05^19^. Remarkably, SJ-05 effectively and selectively degraded PAK4 even with oral administration at 10–30 mg/kg, and in a sarcopenia mouse model, 10 days of oral dosing robustly prevented muscle atrophy. These findings position SJ-05 as a compelling therapeutic candidate and further validate the therapeutic potential of targeting PAK4 through both inhibition and targeted degradation strategies. Future research directions include optimizing selectivity over closely related kinases such as PAK1, evaluating potential toxicities associated with chronic PAK4 inhibition, and comparing therapeutic features with existing medications for metabolic diseases. Finally, determining whether kinase inhibition or targeted protein degradation provides superior efficacy in metabolic and other PAK4-driven diseases remains to be further studied.
